# *ExprEssence *- Revealing the essence of differential experimental data in the context of an interaction/regulation net-work

**DOI:** 10.1186/1752-0509-4-164

**Published:** 2010-11-30

**Authors:** Gregor Warsow, Boris Greber, Steffi SI Falk, Clemens Harder, Marcin Siatkowski, Sandra Schordan, Anup Som, Nicole Endlich, Hans Schöler, Dirk Repsilber, Karlhans Endlich, Georg Fuellen

**Affiliations:** 1 Institute for Biostatistics and Informatics in Medicine and Ageing Research, University of Rostock, Ernst-Heydemann-Str. 8, 18057 Rostock, Germany; 2 Institute for Anatomy and Cell Biology, Ernst Moritz Arndt University Greifswald, Friedrich-Loeer-Str. 23c, 17487 Greifswald, Germany; 3 Department of Mathematics and Informatics, Ernst Moritz Arndt University Greifswald, Jahnstr. 15a, 17487 Greifswald, Germany; 4 Department of Cell and Developmental Biology, Max Planck Institute for Molecular Biomedicine, Röntgenstrasse 20, 48149 Münster, Germany; 5 DZNE, German Center for Neurodegenerative Disorders, Gehlsheimer Str. 20, 18147 Rostock, Germany; 6 Medical Faculty, University of Münster, Domagkstr. 3, 48149 Münster, Germany; 7 Leibniz Institute for Farm Animal Biology, Research Unit Biomathematics and Bioinformatics, 18196 Dummerstorf, Germany

## Abstract

**Background:**

Experimentalists are overwhelmed by high-throughput data and there is an urgent need to condense information into simple hypotheses. For example, large amounts of microarray and deep sequencing data are becoming available, describing a variety of experimental conditions such as gene knockout and knockdown, the effect of interventions, and the differences between tissues and cell lines.

**Results:**

To address this challenge, we developed a method, implemented as a Cytoscape plugin called *ExprEssence*. As input we take a network of interaction, stimulation and/or inhibition links between genes/proteins, and differential data, such as gene expression data, tracking an intervention or development in time. We condense the network, highlighting those links across which the largest changes can be observed. Highlighting is based on a simple formula inspired by the law of mass action. We can interactively modify the threshold for highlighting and instantaneously visualize results. We applied *ExprEssence *to three scenarios describing kidney podocyte biology, pluripotency and ageing: 1) We identify putative processes involved in podocyte (de-)differentiation and validate one prediction experimentally. 2) We predict and validate the expression level of a transcription factor involved in pluripotency. 3) Finally, we generate plausible hypotheses on the role of apoptosis, cell cycle deregulation and DNA repair in ageing data obtained from the hippocampus.

**Conclusion:**

Reducing the size of gene/protein networks to the few links affected by large changes allows to screen for putative mechanistic relationships among the genes/proteins that are involved in adaptation to different experimental conditions, yielding important hypotheses, insights and suggestions for new experiments. We note that we do not focus on the identification of 'active subnetworks'. Instead we focus on the identification of single links (which may or may not form subnetworks), and these single links are much easier to validate experimentally than submodules. *ExprEssence *is available at http://sourceforge.net/projects/expressence/.

## Background

The pace of data generation in the life sciences is steadily increasing. Primary data sets grow in depth and accuracy, covering more and more aspects of life. In molecular biology and biomedicine, these include large-scale measurements of DNA/Histone acetylation, transcriptional activity, gene expression and protein abundance (e.g. [1]). Measuring epigenetic patterns (DNA methylation, DNA/Histone acetylation) on a large scale has become possible only recently [1,2]. Measuring transcription is entering a new era with the introduction of deep (or next-generation, RNA-seq) sequencing [3,4]. Proteomics is becoming possible at unprecedented depth, covering ever-larger parts of the proteome on a routine basis [5]. For these primary data, repositories such as the Gene Expression Omnibus database (GEO [6]) or ArrayExpress [7] are constantly expanding.

Often, measurements are differential: they are made for two or more conditions (such as gene knockdown or knockout [8]), for two or more time points (such as time series tracking the consequences of some experimental intervention, [9]), or for two or more species (such as mouse and human, [10]). *Exploiting differential measurements is one key to cope with the flood of data, by focusing on the most pronounced differences*.

Life scientists also have to handle a deluge of secondary data, in the form of papers, reviews and curated databases. These may be integrated by automated systems such as STRING [11], or by manual efforts [12-14]. *Exploiting secondary data provides another key to cope with the flood of primary data, by putting them into context and focusing on the most pronounced confirmations and contradictions to what is known already*.

In this paper, we propose to interpret differential data in the context of knowledge, yielding the 'essence' of an experiment. Differential data may be provided by two microarrays, and knowledge may be provided by a network describing gene/protein interaction and regulation. In this case, data tracking gene expression in the course of an experiment can be used to identify the most pronounced putative mechanisms. They are identified as those known links between genes/proteins along which expression changes indicate that there may have been some regulatory change, such as the startup or shutdown of an interaction, a stimulation or an inhibition. *ExprEssence *highlights these links, and it enables the user to filter out all links with no or negligible change. The higher the filter threshold on the amount of change to be displayed, the fewer links are shown, making it straightforward to examine the 'essence' of the experiment. Network condensations are illustrated by pairs of figures (original network - condensed network) in the section on Case Studies. The condensed network contains good candidates for interpreting the experiment in mechanistic terms, giving rise to the design of new experiments. However, all inferences are hypotheses derived from correlations in the experimental data in the context of the *a priori *knowledge encoded in the network, and it must be kept in mind that correlative data do *not necessarily *entail mechanistic causality. Moreover, the validity of the hypotheses generated by our method will depend on the coverage and correctness of the network, and on the accuracy of the experimental data.

### Related Work

Starting with the pioneering work of Ideker et al. [15], there is a plethora of methods that combine network data with high-throughput data (such as microarrays), in order to highlight pathways or subnetworks, see the excellent recent reviews of Minguez & Dopazo [16], Wu et al. [17] and Yu & Li [18]. Notably, few of these methods are readily available as publicly accessible software packages, plugins or web services (see Table and in [17]). Also, there does not seem to be a gold standard that can be used for validation purposes (see, e.g., Tarca et al. [19] for a recent discussion). Some methods lack validation except for the example for which they were developed for, while others are studied for an array of specific examples. In these cases, strong enrichment in plausible Gene Ontology categories or detection of known pathways or annotations is often used to demonstrate utility, as in [19-25]. We found two articles including a comparison of different subnetwork identification methods. The first one by Parkkinen and Kaski [26] introduces variants of the Interaction Component Model (ICM) method, comparing them to the original ICM method, to a method based on hidden modular random fields (HMoF) [27] and to Matisse [28], using identification of Gene Ontology classes and coverage of protein complexes for two selected data sets (osmotic shock response and DNA damage data) to judge one method over the other. An evaluation of ClustEx [29], jActiveModules [15], GXNA [21] and a simple approach based on fold change can be found in [29], taking identification of gene sets, pathways and microarray targets known from the literature and from the Gene Ontology for comparison.

In general, it is exceedingly difficult to validate the detection of (sub-) networks or (sub-) pathways: these are complex entities, and ultimate experimental validation is impossible because of this complexity: experimentalists are usually limited to investigating only few components in isolation at any given time. Nevertheless, we will compare results of our method with results obtained by *jActiveModules*, in a separate section following the case studies. In contrast, by just highlighting single links in networks, we tackle a more primitive task, but in this case results can be validated directly by experiment, or by identifying corroborative statements in the literature. In particular, as can be seen from our case studies, the single links that we highlight give rise to predictions about single genes and about single one-step mechanisms that can be investigated in isolation. Therefore, we would like to emphasize the direct utility of our focus on single links and genes, complementing the (sub-)network centric view that is usually employed; to the best of our knowledge, the 'single link and gene' focus is not employed by other methods combining network and high-throughput ('omics') data. In fact, we propose a 'winning combination' of 'network'/'omics' and 'classical' biology, using networks and high-throughput data to highlight single genes and links that may then be validated directly by classical molecular biology, as will be demonstrated in our case studies.

As future work, our formula for link highlighting can, however, be *integrated *into current methods for pathway/subnetwork detection, possibly improving these considerably. In particular, no such method treats inhibitions and stimulations in a distinct way, as we do. In particular, we envision that the edge score formula of Guo et al. [20], which is based on measuring co-variance, may be replaced by our formula (see below), emphasizing a different aspect of differential gene expression: While Guo et al. identify *coordinated *changes using their formula, integration of our formula into their framework would identify subnetworks with changes that are *consistent *with an input network of interactions, stimulations and inhibitions. In any case, we wish to stress that for the identification of coordinated changes, correlation coefficients are most suitable. Our approach, however, identifies a different biological message, namely startups/shutdowns of interactions, stimulations and inhibitions, using an input network that is informative about biological relationships such as stimulations and inhibitions.

## Implementation

*ExprEssence *is implemented in Java Standard Edition 6. It is a plugin for Cytoscape [30], an easy-to-install tool for biological network analysis and visualization. Cytoscape is an open source software project and provides basic features such as network layout and modification. Cytoscape can be enhanced for analysis purposes by straightforward installation of plugins.

### Input data

*ExprEssence *analyses are based on a network of genes and/or proteins, in a format readable by Cytoscape, such as cys, sif, xgmml or gpml. It may be imported from databases using web services such as the Pathway Commons Web Service Client or the WikiPathways Web Service Client [31,32] as a 'simplified binary model' (see Fig. Five in [33]) or it may be downloaded directly from the web. Usually, it reflects expert-curated interaction/regulation data concerning a particular signaling pathway or molecular phenomenon.

The network data must follow a simple specification defined by two constraints:

a) Each link (edge) must be typed to represent either an interaction, stimulation or inhibition. It is possible that all links represent physical interactions, as is the case in a pure protein-protein interaction network. Stimulations and inhibitions are directional, whereas interactions can be interpreted to be un-directional as well as bi-directional.

b) For each gene (node) at least two numerical values must be given on which a meaningful comparison can be based. For example, these may be expression values, derived from measurements in two experiments *E*_1 _and *E*_2_.

By default, for better data interchangeability, *ExprEssence *recognizes Systems Biology Ontology terms [34], also included in the activity flow language of the Systems Biology Graphical Notation (SBGN, [35]), for the specification of interaction types. Thus, each link (edge) must include an attribute called *Interactiontype*, whose values can be either *stimulation *(corresponding to SBO:0000170), *inhibition *(SBO:0000169) or *interaction *(SBO:0000231). In the networks discussed in this article, a single node is used for a gene and its protein product, and the exact nature of the links (edges) denoting stimulations, inhibitions and interactions depends on the evidence underlying the link. For example, a stimulation may be due to the modification of one protein by another, but it may also be the transcriptional stimulation of a target gene by a transcription factor.

The differential measurement data used for comparison may be integrated into the network as described in the Cytoscape manual [36]. Usually, integration is accomplished by mapping unique gene/protein identifiers in the data to unique gene/protein identifiers in the network. The measurements may be gene expression values, but they may also denote protein abundance, methylation levels, etc.

If the numerical data result from multiple measurements (replicates), the number of replicates has to be declared for each experiment, and for each experiment and for each node (gene/protein), the mean value and its corresponding variance have to be given. More specifically, for two experiments *E*_1 _and *E*_2 _to be compared, node *A *has either two or four numerical values: If the data consist of a single measurement, for node *A *these are the two values $M_{A}^{E_{1}}$, $M_{A}^{E_{2}}$. If replicates are analyzed, the two values $M_{A}^{E_{1}}$, $M_{A}^{E_{2}}$ are the mean values and the two variances $Var_{A}^{E_{1}}$, $Var_{A}^{E_{2}}$ are also provided. The number of replicates are *n*_1 _and *n*_2_. *ExprEssence *analyses based on replicated measurements, where mean values and variances are used as input, are more reliable than analyses based on single measurements. Specifically, as the variances are used for calculations, feature variation within and between groups is considered and evaluated appropriately. However, also comparisons based on single measurements can be used to suggest underlying mechanisms.

### Identifying change in a network, motivation

For each link in the network we want to measure the amount of change between experiments *E*_1 _and *E*_2_, where 'change' is a modification in the intensity with which one gene/protein may be influencing another gene/protein; depending on the input data, such influence may be direct physical interaction (in the case of proteins), transcriptional stimulation or inhibition. Therefore, for all links connecting two genes/proteins *A *and *B *in the network under consideration, *ExprEssence *uses the measurements $M_{A}^{E_{1}}$, $M_{A}^{E_{2}}$ and $M_{B}^{E_{1}}$, $M_{B}^{E_{2}}$ for the two experiments *E*_1 _and *E*_2 _to calculate a link score proportional to the amount of change from *E*_1 _to *E*_2_. The formulae are given in the next section. The sign of the score corresponds to the direction of change giving a positive score for startups and a negative score for shutdowns. The magnitude of this signed change corresponds to the absolute value of the score. Links with a link score whose absolute value does not exceed a user-defined threshold are deleted from the network. Hence, only those links are kept, where changes (startups or shutdowns) are pronounced.

Following the heatmap metaphor, large measurement values for genes are indicated by red color and small values are indicated by green color. Similarly, links with a positive value of the link score are colored in red and indicate startups. Links with a negative value are colored in green and indicate shutdowns.

**Figure 1:**
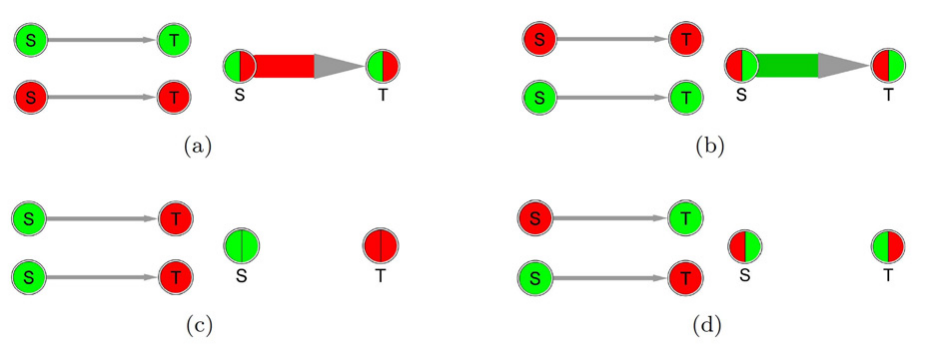
**Network condensation - exemplified for stimulations**. For each of the panels (a) to (d), in the graphs on the left side a stimulator (S) and a target (T) are connected by a stimulation link. Values in the first (*E*_1_, upper graph) and second experiment (*E*_2_, lower graph) are indicated, where low values are marked green and high values are red (following the heat map metaphor). The coloring scheme is red, pale red, white, pale green and green, in order of decreasing values (see text). The graphs on the right side of each panel show the resulting link after applying our method. For each gene, its values for *E*_1 _and *E*_2 _are now inlineed simultaneously in the circle (value of *E*_1 _on the left, of *E*_2 _on the right side). The link connecting both nodes describes the direction and the amount of change between *E*_1 _and *E*_2_. Links with startup of stimulation are colored in red (a), shutdown links in green (b). If values do not change for both the source and the target, or if they change in a completely inconsistent way, the link is removed, see (c) and (d).

**Figure 2:**
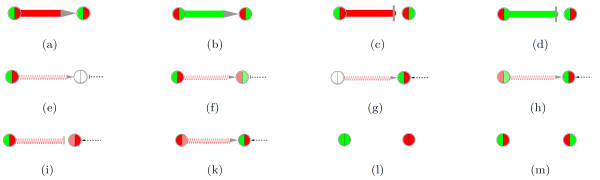
**Network condensation - a gallery of various scenarios**. For each gene, its (expression) values are represented by color. For each link, its score is represented by color and thickness. The coloring scheme is red, pale red, white, pale green and green, in order of decreasing values (see text). Links connecting genes with measurement values changing in an inconsistent way are marked by wavy lines. As in Fig. 1, if the interacting genes are linked by a stimulation *S *→ *T *, the stimulation is assumed to start up, if for both genes, the values go up from *E*_1 _to *E*_2 _((a), *E*_1 _value: left side of circle, *E*_2_: right side of circle), and it is assumed to be shut down if both values go down (b). An inhibition *I * ⊣ *T *is assumed to start up, if the inhibitor value goes up, but the target value goes down (c); it is assumed to shut down if the inhibitor value goes down and the target value goes up (d). In cases (e) and (f) the startup of the stimulation as presented is still a justified hypothesis, even though the target does not go up. For example, in (e) and (f), the stimulation by the source (the stimulator) goes up but it may be counteracted by other inhibiting effects (dashed T-Bar arrow) on the target, as the target does not change (e) or even goes down slightly (f) (*source principle*, see text). In cases where the amount of the stimulator is constant (g) or goes down slightly (h), the startup of a stimulation is still a justified hypothesis based on the target value. Strictly speaking, we hypothesize the startup of the stimulatory *effect *on the target gene. For example, in (g), the startup is not concluded from the change in the value of the stimulator, but it may be due to stimulator accumulation in time, and/or due to cooperation of the stimulator with other stimulations of the target which go up at the same time; startup of the stimulating effect is concluded from the behaviour of the target (*target principle*, see text). Scenario (i) is another example of the *source principle*: it is a justified hypothesis that the inhibition starts up because the amount of inhibitor increases, even though counteracting stimulations drive the amount of the target. Scenario (k) is another example of the *target principle*: it is a justified hypothesis that the stimulatory effect goes up, observing the target and assuming other cooperating effects on it. Lastly, if values do not change at all, or if they change in a completely inconsistent way, the amount of change is zero or near to zero, as in (l) and (m) (also see Fig. 1 (c) and (d)). *Note that cases (e)-(m) all result in reduced link scores. Hence, inconsistent links tend to be removed from the network, as the link score threshold is made more stringent*.

More specifically, in case of a stimulation of gene/protein *T *(target) by gene/protein *S *(stimulator), abbreviated *S *→ *T *, we suppose that the stimulation starts up (from *E*_1 _to *E*_2_), if the values of both genes increase (see Figure 1(a) and Figure 2(a); values for both genes *S *and *T *go up, green to red). If the values of both genes decrease, we suppose that the stimulation shuts down (Figure 1(b) and Figure 2(b)). *In short, we reward correlated change*. In case of an inhibition of gene/protein *T *(target) by gene/protein *I *(inhibitor), abbreviated *I *→ *T *, we suppose that the inhibition starts up (from *E*_1 _to *E*_2_), if the value of the inhibitor increases from *E*_1 _to *E*_2_, and the value of the target goes down (Figure 2(c)). If the value of the inhibitor decreases from *E*_1 _to *E*_2_, and the value of the target goes up, we suppose that the inhibition is shut down (Figure 2(d)). *In short, we reward anti-correlated change*. Other cases, such as no change of values or an inconsistent change, that is an anticorrelated change in case of a stimulation or a correlated change in case of an inhibition, give rise to a link score with a reduced absolute value, see Figure 1(c) and 1(d), Figure 2(e)-(m), and below.

Note that stimulations are treated in a symmetrical way: *S *→ *T *is treated the same way as *T *→ *S*. Indeed, we do not and cannot distinguish *S *→ *T *and *T *→ *S*, because in both cases we expect increments in *S *to be correlated with increments in *T *: Higher amounts of the stimulator go hand in hand with higher amounts of the target. A similar argument holds for decrements. Motivated by this argument, interaction links (*S *↔ *T *) are treated in the same way as stimulation links. This makes sense in general, because the amount of *A *and *B *interacting with each other increases in proportion to the amount of both interactors. More generally, if the interaction represents a biochemical reaction, a straightforward interpretation of our reasoning is given by the law of mass action, see the next section 'Calculation of the amount of change'.

### Calculation of the amount of change

Recall that for measurements of two experiments *E*_1 _and *E*_2_, and two genes/proteins *A *and *B*, we denote the mean of the measured values for *A*, or, if only data of one measurement exists, the single value for *A *in experiment *E*_1 _by $M_{A}^{E_{1}}$, and in experiment *E*_2 _by $M_{A}^{E_{2}}$, respectively. The values for *B *are $M_{B}^{E_{1}}$ and $M_{B}^{E_{2}}$. We can then calculate the amount of change as described in the following. For gene/protein *A*, we determine the differential of *A*, *D_A_*, that is the difference of the measured values between experiments *E*_1 _and *E*_2_:

(1) $$D_{A}=M_{A}^{E_{2}}-M_{A}^{E_{1}}.$$

In case of replicates, *D_A _*is corrected for the variance within the replicates for both experimental conditions, employing Welch's formula [37]:

(2) $$D_{A}=\frac{M_{A}^{E_{2}}-M_{A}^{E_{1}}}{\sqrt{\frac{Var_{A}^{E_{1}}}{n_{1}}+\frac{Var_{A}^{E_{2}}}{n_{2}}}},$$

where $M_{A}^{E_{1}}$, $M_{A}^{E_{1}}$: Mean value of gene/protein *A *under experimental condition *E*_1_, *E*_2_;

$Var_{A}^{E_{1}}$, $Var_{A}^{E_{1}}$: Variance of values of gene *A *under experimental condition *E*_1_, *E*_2_;

*n*_1_, *n*_2_: Number of replicates done in experiment *E*_1_, *E*_2_.

*D_B _*is determined analogously. This equation corresponds to the Welch t-test for comparison of mean values of two samples with unequal variances. As we do not want to make strong preconditions about the statistical distribution of the samples, we do not calculate p-values. The weaker preconditions for Welsh's t-test are fulfilled if, for both experiments, independent samples are measured, and if their values are approximately normally distributed. Given *D_A _*and *D_B_*, the amount of change for an interaction link is the sum of the two differentials:

(3) $${\text{LinkScore}}_{Int}=D_{A}+D_{B}.$$

Taking the difference $M_{A}^{E_{2}}-M_{A}^{E_{1}}$ and not $M_{A}^{E_{1}}-M_{A}^{E_{2}}$ reflects the motivation to denote startups of interactions by a positive score and shutdowns by a negative score.

The formula gives scores with high absolute value for correlated changes of the values of *A *and *B *from *E*_1 _to *E*_2_. Depending on the direction of the correlated change, the score becomes positive or negative which denotes a startup or shutdown of the interaction/stimulation. Anti-correlated changes are given a reduced absolute value of the score (see below and Figure 2(f) and 2(h)). The formula is simple, yet powerful:

1. In the specific case of a physical interaction between two proteins, and *log*-transformed data, the formula above corresponds to the law of mass action, as follows. The 'activity' of a physical interaction of protein *A *with protein *B *can be expressed by the product of the abundances of both, assuming that the expression values correspond to the 'amount' of protein. The 'amount' of the complex *AB *in experiment 1 can then be compared to the 'amount' of the complex *AB *in experiment 2, by taking the ratio. Large changes in this ratio indicate that there will be much more or much less of the protein complex, comparing experiment 1 with experiment 2. (Note that we do neither calculate equilibrium constants nor reaction kinetics.) As we have two experimental conditions and are interested in the change from *E*_1 _to *E*_2_, startup of 'activity' is thus proportional to the ratio of the products of the abundances of *A *and *B*, taking experiment *E*_2 _over experiment *E*_1_: $\left({\left[A\right]}^{E_{2}}\cdot \;{\left[B\right]}^{E_{2}})/({\left[A\right]}^{E_{1}}\cdot {\left[B\right]}^{E_{1}}\right)$. In case of *log*-transformed values, this is the difference of the sums of the measurement values under both conditions: $\left(M_{A}^{E_{2}}+M_{B}^{E_{2}})-(M_{A}^{E_{1}}+M_{B}^{E_{1}}\right)$. This can be written as $\left(M_{A}^{E_{2}}-M_{A}^{E_{1}})+(M_{B}^{E_{2}}-M_{B}^{E_{1}}\right)$ and corresponds to *D_A _*+ *D_B _*from formula (3). Hence, our formula for the link score of interaction links can be connected directly to the law of mass action.

2. As explained above, we can treat the stimulation of a gene/protein *A *by a gene/protein *B *in the same way as an interaction of the two proteins with each other and therefore use the same formula to determine the link score:

(4) $${\text{LinkScore}}_{Stim}={\text{LinkScore}}_{Int}=D_{A}+D_{B}.$$

3. Formula (4) can be modified to capture inhibitions *A *→ *B *(*A *inhibits *B*), where *A *and *B *are expected to be anticorrelated in their expression/amount:

(5) $${\text{LinkScore}}_{Inh}=D_{A}-D_{B}.$$

This equation honors the case where higher amounts of the inhibitor *A *go hand in hand with lower amounts of the target *B *and vice versa, whereas correlated changes are penalized (see Figure 2(c) and 2(d)).

4. Our formulae deliver justified hypotheses also in the cases that are not as straightforward as the cases in Figure 1(a)-(b)/Figure 2(a)-(d), given two additional assumptions, that we call the *source principle *and the *target principle*. It is important to note that these complicated cases are characterized by relatively low link scores and additionally they will be marked by wavy lines. Furthermore, they can be identified by inspecting the color-coded measurement values (Figure 2(e)-(k)), which can be made explicit by addition of gene/node labels as in the condensed networks of case studies 2 and 3.

The *source principle *maintains that changes in the source, if they are large enough, are sufficient for a hypothesis regarding startup/shutdown of a stimulation/inhibition. Even if the value of the target is inconsistent, putting trust into the network data (that is, the stimulation/inhibition link is not questioned), the link then describes a startup/shutdown which is assumed to act on the target, even though it is counteracted by other effectors (Figure 2(e),(f),(i)). The other effectors may or may not be included in the network: we assume that the network is correct, but not necessarily complete. In case of transcriptional stimulations/inhibitions, a simple example for counteracting effectors are transcription factors that act in an opposite way at a different position of the regulatory region of the target gene. Here, we view gene regulation as a 'transcription factor battlefield' [38,39]. In fact, the target gene may not be observable (expressed) at all without the stimulation that is highlighted. There is alternative interpretation for an inconsistent target value: The stimulation may not be in the scope of what is being measured. For example, if the values refer to expression levels, a stimulation of the target by phosphorylation goes undetected.

The *target principle *holds that large changes in the target are sucient for a hypothesis regarding startup/shutdown of a stimulation/inhibition, even if the value of the source (the stimulator/inhibitor) is inconsistent. Again trusting the network data, the link then describes a startup/shutdown that is becoming relevant because other effectors are now cooperating on the target (Figure 2(g),(h),(k)). Then, strictly speaking, in all these three cases we hypothesize that it is not the stimulation itself that goes up, but its effect on the target gene. Again, we view gene regulation as a 'transcription factor battlefield'. Also, the other effectors may or may not be part of the network. Of course, the inconsistent change in the source has to be lower than the tale-telling change in the target. Also, the startup of the stimulating effect is assumed to require only a low amount of the stimulator, which is however still exceeded. There is an alternative interpretation for an inconsistent source value: The stimulating effect may simply be delayed in case of a time series, where the stimulator (protein) needs time to accumulate, which may also happen during a period of constant or down-regulated gene expression of the stimulator.

Naturally, inconsistencies can also give rise to revision of the network. However, our formula is not designed to reveal severe inconsistencies (since such links receive scores close to zero and are removed from the network as in Figure 2(m)).

5. To distinguish straightforward from inconsistent cases by inspection, and to aid the interpretation of links, our plugin offers multi-colored nodes, inlineing directly the measurement values of a gene for a pair of experiments within a single node as a pie-chart as explained in Figure 1 and inlineed in Figure 2. To calculate the color for visualization of the values in the pie-chart, we take the 10%, 50% and 90% quantiles of the ordered list of all attribute values. The value associated with the 10% quantile defines the lower threshold. All values below this threshold are visualized by green color of same intensity. Values above this threshold and up to the value corresponding to the 50% quantile get a color defined by linear interpolation between the 10% quantile (green color) and the 50% quantile (white). Analogously, values are visualized by a color between white (50% quantile) and red (90% quantile). Values above the 90% quantile are represented by red color of same intensity. The thresholds and the coloring scheme can be redefined by the user. Furthermore, our plugin provides labeling of selected genes/nodes with the measurement data used for node coloring as shown in the condensed networks of case studies 2 and 3.

Finally, depending on the value of the *Interactiontype *attribute for a link, the respective formula for the link score is as follows:

(6) $$LinkScore=\{\begin{array}{ll} {\text{LinkScore}}_{Int} & \text{if}\;Interactiontype=\;Interaction,\\ {\text{LinkScore}}_{Stim} & \text{if}\;Interactiontype=\;stimulation,\\ {\text{LinkScore}}_{Inh} & \text{if}\;Interactiontype=inhibition. \end{array}$$

We will use this link score to identify those links along which there is a large change between *E*_1 _and *E*_2_. Links with a link score exceeding a user-defined threshold are colored in red or green; the other links are deleted from the network.

### Condensation of networks

After importing the network and measurement data into Cytoscape, the *ExprEssence *dialog window is used to define which data shall be taken for calculation of the link score and hence for network condensation. As discussed above, the network must include at least two numerical attributes for each gene/protein, so that the formulae can be employed. These two attributes are explicitly selected by the user, indicating their order (*E*_1 _versus *E*_2_, or *E*_2 _versus *E*_1_). After selecting two attributes, the user may then indicate that there is variance data available and specify the number of replicates. In this case, the measured values are implicitly assumed to be the mean values for which the variances are provided. Finally, calculations are started and results are inlineed in a new network window in Cytoscape. Links with a positive change (startups) are rendered in red, and negative change (shutdown) is rendered in green. Color saturation and link thickness are directly linked to the link score calculated.

**Figure 3:**
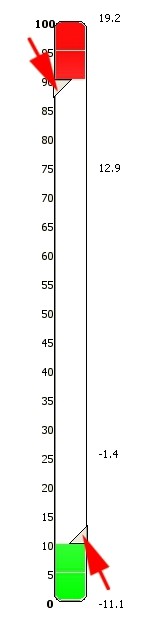
**Slider in the *ExprEssence *user interface**. Thresholds may be set independently for both the upper (red arrow) and the lower (green arrow) side of the spectrum of the link scores.

In the user control interface of *ExprEssence*, a slider (Figure 3) is provided to define the threshold to keep all links with link score exceeding the threshold, on both the positive (startup) and negative (shutdown) side of the spectrum of link score values. Using this slider, the user can cut the number of links in the network. The more stringent the threshold, the more links are removed and only links with high absolute value of the link score will remain. Genes which have no link left after removal of links are also removed from the network. Using the condensed network, the user can investigate components of the network where interactions, stimulations or inhibitions start up or shut down, comparing experiment *E*_1 _with *E*_2_.

## Results

We present results of the application of *ExprEssence *in three case studies.

### Case Studies

We will describe three application scenarios, condensing networks and describing the insights gained from these. As a first example, we condense a network based on literature-curated interaction data of proteins involved in structure and function of the podocyte, which is the cell forming the kidney filtration barrier. The second example will describe how a hand-curated network of interaction and regulation of genes maintaining the pluripotent state of stem cells can be condensed using microarray data tracking an early transition process of embryonic stem cells, yielding a mechanistic hypothesis that was then confirmed experimentally. In a third application, we will take a biological network describing ageing-related processes from the WikiPathways database, integrate publicly available microarray data, and confirm some basic insights into ageing. Cytoscape session files PodocyteCellMatrix.cys, Epiblast.cys and DNA_Damage.cys are provided as Additional Files 1, 2 and 3, and they enable reproduction of figures following the instructions given there.

#### Case Study 1 - Interaction network of podocyte cell-matrix proteins

Podocytes cover the outer aspect of the capillaries in the kidney glomerulus, where the ultra filtration of blood takes place. The filtration barrier is composed of endothelial cells, the glomerular basement membrane (GBM) and podocytes. The proper function of podocytes is essential for the ultrafiltration process. Podocytes synthesize the majority of extracellular matrix molecules that are present in the GBM. The podocyte-GBM interface is crucial for mechanical anchorage and inside-out as well as outside-in signaling. Damage or loss of podocytes is estimated to be responsible for about 90% of kidney diseases in humans [40]. To date several hereditary kidney diseases are known that are caused by mutations in genes involved in the podocyte-GBM interface, e.g. Alport syndrome. Thus, the podocyte-GBM interface is of central importance in kidney biology and pathology.

We constructed a protein interaction network of the podocyte-GBM interface based on expert knowledge.

**Figure 4:**
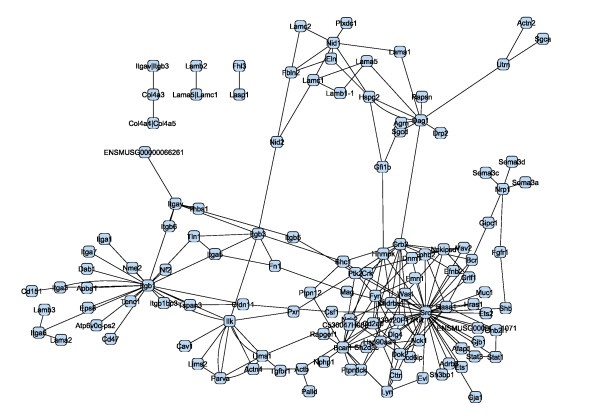
**Protein interaction network of the podocyte GBM-interface (STRING-extended expert network)**.

We collected proteins and experimentally well-described protein-protein interactions of the podocyte-GBM interface by a comprehensive survey of the podocyte literature. The expert network consists of 42 nodes (proteins) and 33 edges (protein-protein interactions). The proteins of the expert network were screened for further interaction partners utilizing the STRING database [11], to extend the expert network by further experimentally verified interactions involving at least one node (protein) of the network. If not yet existent in the network, the respective interaction partners were also added. The extended network consists of 124 nodes and 206 edges (Figure 4).

Podocyte cell lines are a frequently employed tool to study podocyte biology. However, it is well known that podocyte cell lines are partially dedifferentiated as compared to *in vivo *podocytes. To extract the main differences between the podocyte-GBM interface of *in vivo *vs. cultured podocytes, we mapped microarray gene expression data of *in vivo *and cultured mouse podocytes onto the extended network shown in Figure 4. We used publicly available microarray data (GSE10017, [41]) generated from a podocyte cell line and from *in vivo *podocytes, which were isolated as podocalyxin-positive cells in a cell suspension of enzymatically digested mouse glomeruli. By condensing a protein interaction network using gene expression data, we implicitly assume that protein abundance is correlated to gene expression. We *log*-transformed and quantile-normalized these data.

**Figure 5:**
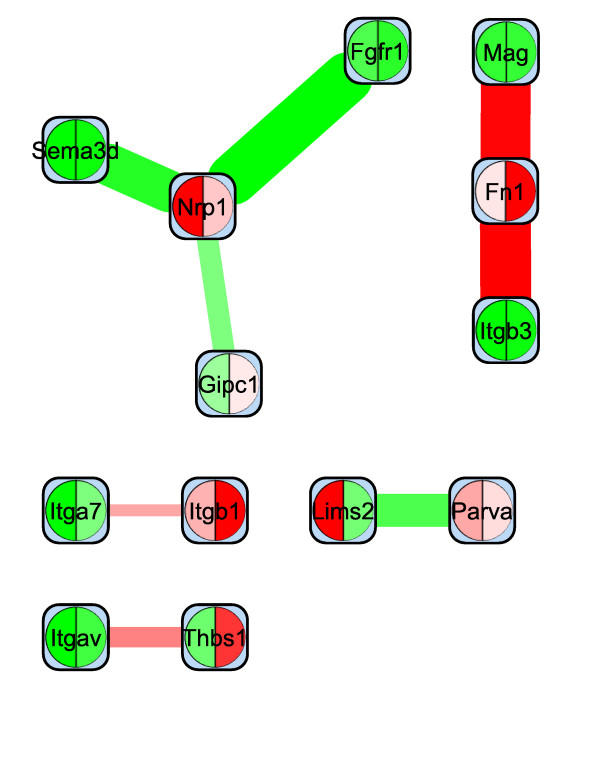
**Condensed network resulting from the application of *ExprEssence *to the network in Fig. 4, using gene expression data from *in vivo *(left side) and cultured podocytes (right side)**. Only links with highest and lowest link score were kept (3% quantile on both sides).

**Figure 6:**
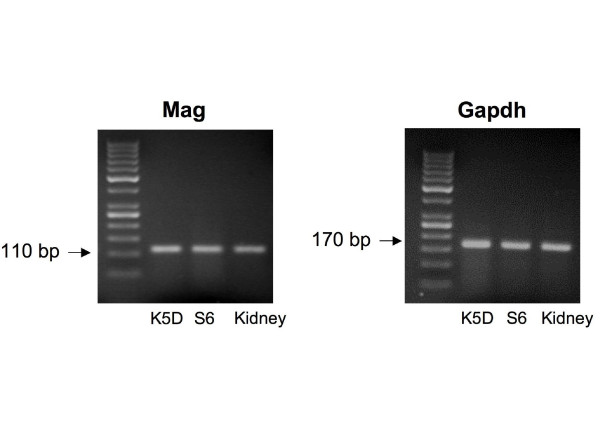
**Podocyte RT-PCR expression analysis of Mag**. Mouse kidney and two mouse podocyte cell lines (K5 D and S6) were cultured as reported earlier [63]. Podocyte RNA was isolated using a mixture of guanidine thiocyanate and phenol (TRI Reagent, Sigma) according to the manufacturer's protocol. Reverse transcription was performed on 5 *μ*g denaturated RNA. Real-time PCR was performed with a Light Cycler (Roche Diagnostics, Mannheim, Germany) using the Platinum^® ^SYBR^® ^Green qPCR SuperMix-UDG kit (Invitrogen, Heidelberg, Germany) and run at 95°C for 10 min followed by 45 cycles at 95°C for 10 s, 60°C for 5 s, and 72°C for 12 s. Relative expression was normalized using GAPDH. The following primers were used: Mag sense 5'-TGG GCC TAC GAA ACT GTA CC-3', anti-sense: 5'-GCT CCG AGA AGG TGT ACT GG-3', 110 bp expected product size; Gapdh sense 5'-ACC CAG AAG ACT GTG GAT GG-3', antisense 5'-CAC ATT GGG GGT AGG AAC AC-3', 170 bp expected product size. A 50 bp DNA step ladder was loaded in the left lanes.

By interactive use of *ExprEssence *we removed 94% of the edges keeping the 3% quantiles of the most strongly differentially altered interactions between in vivo and cultured podocytes (Figure 5). *ExprEssence *revealed that the interactions of semaphorin 3 d (Sema3d), fibroblast growth factor receptor 1 (Fgfr1) and Gipc1 PDZ domain-containing protein (Gipc1) with neuropilin 1 (Nrp1) as well as the interaction between pinch 2 (Lims2) and *α*-parvin (Parva) are most strongly diminished (green links) in cultured podocytes as compared to the *in vivo *situation. On the other hand, the interactions of integrin *β*_3 _(Itgb3) and myelin-associated glycoprotein (Mag) with fibronectin 1 (Fn1) are most strongly up-regulated in cultured podocytes. As Mag had so far not been reported as a podocyte protein, we analyzed Mag expression by RT-PCR in a podocyte cell line. Indeed, Mag expression was easily detected in cultured podocytes (Figure 6), revealing a novel candidate for the podocyte-GBM interface.

Podocytes dedifferentiate under cell culture conditions. Dedifferentiation of podocytes in culture may recapitulate dedifferentiation of podocytes *in vivo *during kidney disease. Thus, comparing gene expression between cultured and *in vivo *podocytes may give important clues about essential proteins and protein interactions needed for proper podocyte function. *ExprEssence *segregates the most strongly differentially altered interactions between cultured and *in vivo *podocytes, corroborating previous findings and discovering novel protein interactions that might be involved in the podocyte-GBM interface:

1. Pinch and parvin participate in integrin signaling via integrin-linked kinase. This pathway is essential for podocyte function, since mice with podocyte-specific knockout of integrin-linked kinase die from renal failure at the age of 16 weeks [42]. The pinch/parvin interaction is shut down in cultured podocytes (see Figure 5), making it a candidate key interaction reflecting podocyte dedifferentiation in cell culture. In the healthy kidney, pinch and parvin may have an important role in transmitting signals from the extracellular matrix through integrin-linked kinase, to maintain podocytes in a differentiated state [43].

2. Neuropilin and its interaction with the guidance molecule semaphorin have been implicated in podocyte differentiation [44,45]. The interaction of neuropilin with several proteins, including semaphorin, is greatly diminished in cultured podocytes (see Figure 5). *ExprEssence *uncovers that loss of neuropilin interaction with extracellular molecules also participates in the dedifferentiation of podocytes in culture as suggested by the *in vivo *findings [46].

3. Massive up-regulation in cultured (= dedifferentiating) podocytes of the interaction between fibronectin 1 and the membrane protein Mag, suggest an important and hitherto unknown function of Mag in the regulation of podocyte differentiation through the podocyte-GBM interface. Indeed, we could confirm podocyte expression of myelin-associated glycoprotein (Mag) (Figure 6), which has so far not been implicated in podocyte biology. Since myelin proteins are known to be expressed only in glial cells of the nervous system, it is also notable that knockout of myelin protein zero, another myelin protein preferentially expressed in podocytes within the glomerulus, has been shown to result in proteinuria [47].

#### Case Study 2 - Analysis of a pluripotency-related experiment

**Figure 7:**
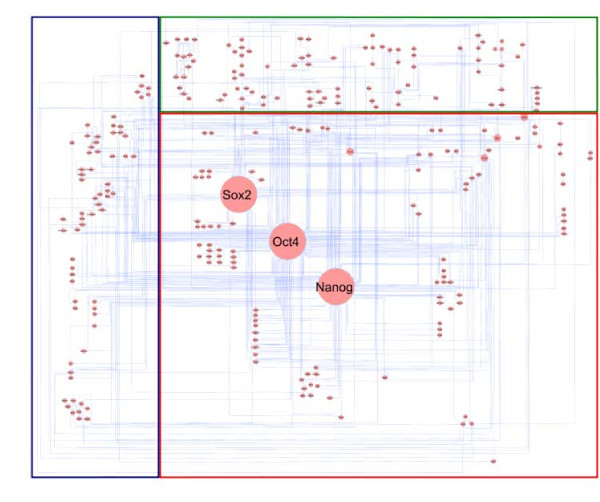
**A network describing pluripotency-related interaction and regulation data assembled from the literature**. The core part describes gene regulation, the upper part signaling pathways, and the part on the left epigenetic phenomena.

Stem cell research is currently one of the most active areas in molecular biology and biomedicine, based in part on recent breakthroughs in generating 'induced pluripotent stem cells' (iPS cells) from somatic cells like fibroblasts (reviewed in [48,49]). Such a 'reprogramming' of differentiated cells into 'pluripotent' ones is possible by directly manipulating gene regulation in the cell, confronting the differentiated cell with artificial amounts of key transcription factors such as Oct4 (also known as POU5F1), Sox2 and Nanog. These 'ectopic' factors then re-direct the overall network of interaction and regulation into a direction that is so close to the 'embryonic state' that mice can be obtained, in which some (or even all) of their cells derive from the manipulated somatic cells [50]. A mouse suering from sickle-cell anemia was healed by reprogramming fibroblasts from its tail, correcting the genetic defect, and re-differentiating the iPS cells into blood-building cells that were then injected [51]. In human, iPS technology already allows to study a patient-specific disease in the 'petridish', and to regenenerate tissues by re-differentiating iPS cells. Safety concerns currently hinder the engraftment of 'healed' tissue, and triggering the re-direction of the regulatory network by chemical compounds is one avenue to improve safety. Consequently, molecular analyses of the induction of pluripotency and of (re-)differention triggered by small chemical compounds is of high interest in the human as well as in the mouse system. Over the past year, we have assembled a network of molecular interactions, stimulations and inhibitions from 135 publications until March 2010, involving 262 genes/proteins of mouse. The network includes the core circuit of Oct4, Sox2 and Nanog, its periphery (such as Klf4, Esrrb, and c-myc), connections to upstream signaling pathways (such as Activin, Wnt, Fgf, Bmp, Insulin, Notch and LIF), and epigenetic regulators (Figure 7). An updated (June 2010) version of this 'PluriNetWork' is described in [14].

**Figure 8:**
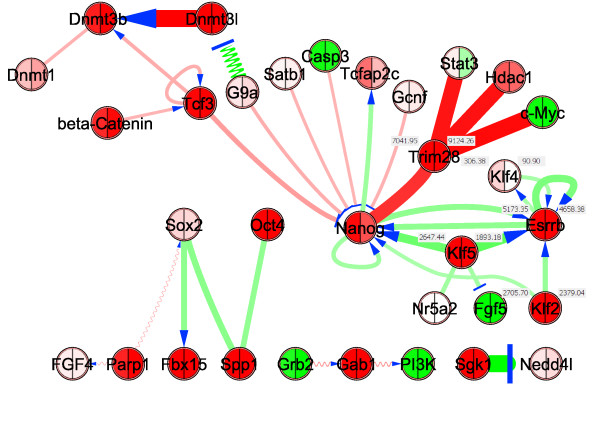
**Network of protein interactions and gene/protein stimulations and inhibitions involved in pluripotency, condensed using *ExprEssence *and microarray data tracking the transition process from embryonic stem to epiblast-like cell state**. Expression values for Trim28, Esrrb, Klf4, Klf2, Klf5 are inlineed to the left (ES state) and to the right (epiblast state) of the gene (node). Esrrb and Trim28 are both found in the upper right corner.

**Figure 9:**
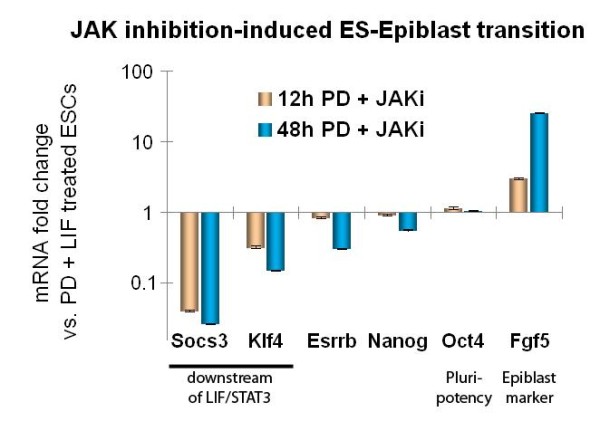
**Esrrb down-regulation is a later event in the ES-Epiblast transition**. Real-time PCR analysis of mouse embryonic stem (ES) cells treated for 12 and 48 hr with a pharmacological inhibitor against JAK to inhibit LIF/JAK/STAT3 signaling, which induces a partial transition to the epiblast state. Note that the known LIF/STAT3 target genes Socs3 and Klf4 are rapidly down-regulated at 12 hr. At this timepoint, Esrrb expression is almost unaffected. As suggested by the model, however, Esrrb inlines down-regulation at 48 hr, whilst Oct4 expression is stable (the FGF/MEK/ERK-inhibitor PD (PD0325901) was applied in all experiments).

Applying *ExprEssence *to our expert network, we analyzed recently published data (GSE17136 [52]) on the effect of a pharmacological inhibitor (JAKi, Janus kinase Inhibitor I, Merck) on embryonic stem cells, which triggers a transition process from the embryonic stem cell to another pluripotent cell state, the epiblast stem cell state. The effect is described by microarrays taken before, and 12 hours after the intervention. We kept the 5% quantiles of links with the largest amount of change. We observed that shutdown of stimulations is centered around the protein Esrrb, the expression of which is just slightly diminished (see Figure 8). Cooperative Esrrb regulation by a variety of transcription factors such as Klf4, Klf2 and Klf5 has already been observed by Jiang *et al. *[53]. Thus, we predict Esrrb down-regulation at a later time point. More specifically, Figure 8 inlines the condensed expert network, describing the effects of inhibition of the LIF/Jak/Stat3 signaling pathway [52] by the JAK inhibitor I. Notably, the stimulations of Esrrb by Nanog [54], Klf2, Klf4, Klf5 [53] and by itself [55] are shut down. These shutdowns are the result of down-regulation of these stimulators within the first 12 hours. Klf2, Klf4, Klf5 and Nanog are known to be upstream of the ES cell-specific transcription factor Esrrb [53,55,56]. However, a strong effect on Esrrb was not yet seen at the 12 hour time-point, but according to Figure 8 our model suggested a down-regulation of Esrrb as a consequence of JAK inhibitor-mediated down-regulation of its upstream factors. To test this hypothesis, we carried out real-time PCR analysis of JAKi-treated ES cells at a later time-point, 48 hours. As can be inferred from Figure 9 Klf4 was already down-regulated at 12 hours but its downstream target gene Esrrb was not. At 48 hours, however, we did observe significant down-regulation of Esrrb, confirming the idea of its shutdown via other members of the ES cell self-renewal network.

As Klf4 and Nanog are known to be stimulated by Esrrb [55,56], these stimulations are also shut down (Figure 8*target principle*). Finally, interactions between the transcription factors Stat3, Hdac1, c-Myc & Nanog and Trim28 (also known as TIF1*β*, a transcription co-regulator (co-repressor) and chromatin modifier [57,58]) are started. These startups are highlighted because the Trim28 expression value goes up strongly, from 7041 to 9124. The role of these startups is unknown, though they may reflect the general repression of components of the ES cell-specific self-renewal network by Trim28.

#### Case Study 3 - Analysis of ageing-related experiments

**Figure 10:**
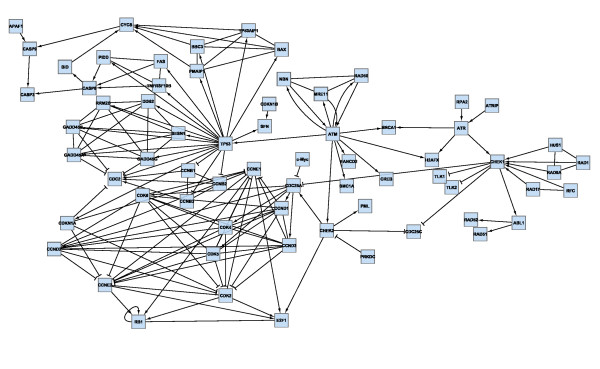
**Network adapted from the WikiPathways 'DNA damage response' network in human**.

**Figure 11:**
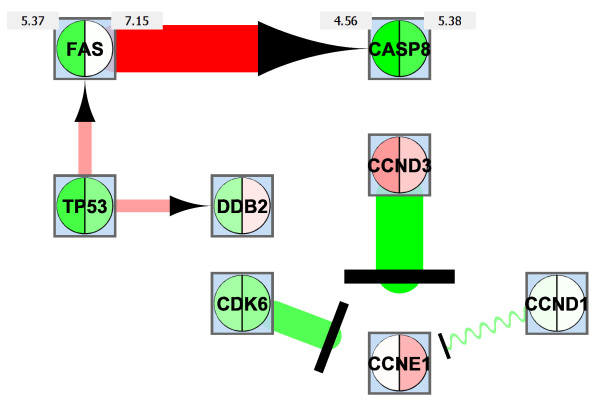
**Condensed network resulting from the network in Fig. 10**. The genes (nodes) contain color-coded (heat map) information about gene expression among young subjects (left side) and old subjects (right side). Expression values for FAS and CASP8 are inlineed to the left and right of the gene (node).

To study the effects of ageing on DNA damage response, we retrieved a network from WikiPathways [31], 'DNA damage response' in human, as of May 22, 2010. After importing it to Cytoscape, we expanded all complexes yielding the network in Figure 10. For example, for a complex in the original network such as CDK2, CCNE1 and CCNE2, all genes were connected pairwise to each other. We then integrated *log*-transformed and quantile normalized microarray data from GSE11882 [59]. From this dataset we used only the data obtained from the hippocampus. We considered the same four age categories (20-39, 40-59, 60-79, and 80-99 years) as in [59]. Using *ExprEssence*, we analyzed the changes between the first (20-39 years) and the last age category (80-99 years) and kept the 3% quantiles of the most strongly differentially altered links. The startup of the stimulation of CASP8 by FAS (Figure 11 top red link) and the shutdown of the inhibition of CCNE1 by CCND3 are the largest changes. The up-regulation of apoptosis, highlighted by the red link between FAS and CASP8 just mentioned, is the result of stimulation by p53 (TP53), and is a known phenomenon in ageing processes [60]. Note that the expression value of CASP8 is going up slightly (from 4.56 to 5.38), whereas the up-regulation of FAS is more pronounced (from 5.37 to 7.15). The down-regulation of the inhibition of CCNE1 by CCND3 [61] and CCND1 as well as by their corresponding kinase CDK6 may trigger the higher expression of CCNE1, indicating a deregulation of the cell cycle. Finally, we found ageing-related up-regulation of a DNA repair pathway, that is, stimulation of DDB2 by p53 [62].

### Subnetwork identification by *jActiveModules *for Case Studies 1-3

To put the results obtained in case studies 1-3 into the context of related work, we used *jActiveModules *[15] to analyze the same data, identifying 'active modules', that are subnetworks where the constituent genes show significant changes in expression over the two conditions we investigate. As discussed in the section on 'Related Work', the aim of *ExprEssence *is quite different, namely the identification of single links (interactions, stimulations, inhibitions) and genes affected in the course of an experiment, where the links do not necessarily have to build up a connected subnetwork. Furthermore, *ExprEssence *exploits the knowledge about stimulations and inhibitions that may be encoded in the network.

We used *jActiveModules *with default parameters. In contrast to *ExprEssence*, which takes two expression values per gene (one for each experimental condition), *jActiveModules *requires one p-value per gene (describing the statistical significance of the expression change between the two experimental conditions; p-values were used as calculated while processing the raw expression data for the case studies).

**Figure 12:**
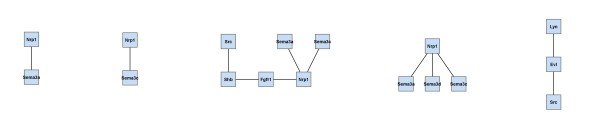
**Results of *jActiveModules*, default parameters, applied to the Podocyte dataset (case study 1)**. See also Figs. 4/5.

Figure 12 inlines the results of *jActiveModules *applied to the data of case study 1 (podocyte cell-matrix proteins). Module scores are (from left to right) 4.048, 3.384, 2.927, 2.861, 2.761. The first four subnetworks are overlapping. The Nrp1 gene/protein that is found in these four subnetworks is also found in our condensed network (Figure 5). In Figure 12 Nrp1 is linked to Sema3a & Sema3c, as well as to Fgfr1 & Sema3 d, and expression of these four genes indeed changes significantly. Links to the latter two genes are highlighted by *ExprEssence*, because change of expression is correlated as in Figure 1(b), even though Fgfr1 & Sema3 d change only slightly. Links to the first two genes are not highlighted by *ExprEssence*, because change of expression is anti-correlated as in Figure 1(d), and the link threshold is not exceeded (Figure 1(d) describes a case of perfect anti-correlation yielding a link score of 0). Similarly, the subnetwork Lyn-Evl-Src is not highlighted, because the link scores are below threshold. In turn, the links between Fn1 and Mag/Itgb3 are not picked up by *jActiveModules*, because Mag/Itgb3 do not change with sufficient significance (p-value); the same holds true for Itga7, Parva and Itgav.

**Figure 13:**
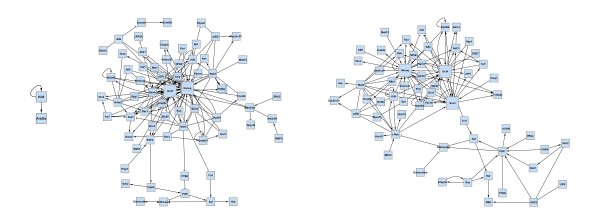
**Results of *jActiveModules*, default parameters, applied to the Pluripotency dataset (case study 2)**. See also Figs. 7/8.

The results of *jActiveModules *for case study 2 (transition from the embryonic stem cell to the epiblast stem cell state) are shown in Figure 13. Interestingly, we discover one small and two very large modules, scoring 3.612, 3.386, 2.768, respectively. The small network is composed of Klf4 (which is also a focus of highlighting by *ExprEssence*, due to its strong down-regulation) and Arid3a, which is the protein linked to Klf4 that changes most significantly. The two large modules have significant overlap with each other, and also with the *ExprEssence*-condensed network (Figure 8), but it can immediately be seen that the latter is more informative than the results of *jActiveModules*, due to link thickness and coloring, allowing easier identification and interpretation of mechanisms behind the observed expression change.

**Figure 14:**
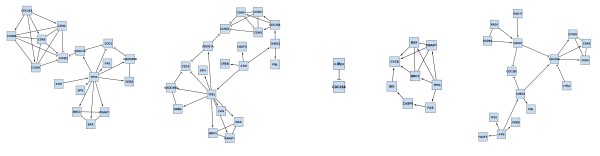
**Results of *jActiveModules*, default parameters, applied to the Ageing dataset (case study 3)**. See also Figs. 10/11.

Finally, we put together the active modules found for the Ageing example of case study 3 (Figure 14). As in case study 2, large overlapping networks are obtained. Module scores are (from left to right) 1.899, 1.868, 1.387, 0.786, 0.547. The majority of the modules identified by *jActiveModules *include the link between TP53 and FAS, which is also highlighted by *ExprEssence*. The link between FAS and CASP8 is only considered marginally active (it is found in one module), because CASP8 does not feature a change with a high p-value. The link between CCND3 and CCNE1 is not considered by *jActiveModules*, because change of CCNE1 is not sufficiently significant.

Overall, we observe an overlap of results between our tool and *jActiveModules*. In all case studies, *jActiveModules *did not identify many of the links/effects on genes that we discovered and validated. However, it identified interesting subnetworks (around Nrp1; Klf4-Arid3a; around TP53) that are plausible and worth investigating. Most importantly, however, *ExprEssence *can distinguish stimulations and inhibitions, and by marking links in thick green or red color, we enable a more informed focus on single links and genes, directly yielding suggestions for experiments that may test the hypotheses we generate.

## Conclusions

The most important limitation of our approach is that highlighting is neither necessary nor sufficient for detecting mechanistic change. More specifically, it is quite possible that no change (no startup or shutdown of an interaction, stimulation or inhibition) happens across a highlighted link, or that change happens across a link that is not highlighted. The main reason for this problem is missing accuracy (in terms of sensitivity, i.e. false negatives, and specificity, i.e. false positives) of both network and measured data. In particular, many networks are seriously incomplete, so that we cannot highlight the 'essential' mechanisms simply because there are no links in the network that represent them. For example, the main mechanism may be mediated by a regulatory RNA, which may be neither represented in the network, nor in the expression data gained by microarray experiments. Then, we simply cannot discover it, and the mechanisms that are highlighted will be either minor, or simply false positive. To give another example, imagine that the network data do not cover a gene *C *that acts on both *A *and *B*, but it includes the link *A *→ *B*. Then, the link may be highlighted even though *C *is acting on both *A *and *B*, and nothing more. Suce it to say, hypotheses generated with the help of *ExprEssence *have to be validated experimentally. On the other hand, in a signaling cascade, the mode of change (information flow) may be via phosphorylation events that cannot be measured by expression data. Then, *A *may stimulate *B *via the link *A *→ *B*, but no change is detectable in the differential expression data, and no highlighting occurs.

With our approach towards identification of the critical parts of a gene/protein network using differential data, we offer a means to easily become aware of changes in gene/protein relationships that can be observed by contrasting two experimental conditions. We do not only consider physical interactions between proteins but are able to take into account stimulations or inhibitions and treat them accordingly in order to get specific insights into regulatory aspects. *ExprEssence *identifies startup/shutdown along all three different link types (interaction, stimulation, inhibition) in a coherent manner. Our method does not depend on a specific type of network or experimental data as long as edges in the network connect entities influencing each other and the experimental data can be interpreted as measurements proportional to the abundance of the entities.

The statistical basis for comparison of link scores of different edges depends on the input data: if no replicates are available, the plugin works without any measurement of variability, and allows exploration of the dataset. If replicates are given, the plugin uses Welch's formula to improve comparability of link scores by considering the variability of the measurements.

Despite its limitations, we developed a simple, straightforward and easy-to-use tool for hypothesis building, towards a mechanistic interpretation of experiments, seeing the forest for the trees in a large amount of data.

## Competing interests

The authors declare that they have no competing interests.

## Authors' contributions

GW wrote the software, CH, MS, AS, GF contributed to software development and testing, GW, DR, MS, KE, GF contributed to method development, GW, BG, SF, NE, KE, GF contributed to data analysis, BG, SS conducted experiments, GF, GW, KE, SF wrote the paper, GF, KE, HS designed and supervised research. All authors read and approved the final version of the manuscript.

## Additional Files

**PodocyteCellMatrix.cys, Epiblast.cys, DNA_Damage.cys**. Cytoscape Session files containing the original network, expression data and condensed network from case studies 1-3.

## Supplementary Material

Additional file 1 **PodocyteCellMatrix.cys**. To reproduce Figure 5 open the file 'PodocyteCellMatrix.cys' in Cytoscape, select the uncondensed network, start *condense!*, select 'Shaw_Ensembl' (left side) and 'Mundel_Ensembl' (right side) and *No variance data*. After submitting, click on *Organic Layout*. Click here for file

Additional file 2 **Epiblast.cys**. To reproduce Figure 8 open the file 'Epiblast.cys' in Cytoscape, select the uncondensed network, start *condense!*, select '12 h PD+LIF Signal' (left side) and '12 h PD+JAKi Signal' (right side) and *No variance data*. Modify the position of the two sliders to select the 5% and 95% quantiles, and choose *Organic layout*. Click here for file

Additional file 3 **DNA_Damage.cys**. To reproduce Figure 11 open the file 'DNA_Damage.cys' in Cytoscape, select the uncondensed network, start *condense!*, select 'HC 1 mean' (left side) and 'HC_4_mean' (right side) and, as variance data, select 'HC_1_var' (left side) and 'HC_4_var' (right side). The number of replicates is 10 (left side) and 16 (right side). After submitting, click on *Organic Layout*. Click here for file
